# Rural–Urban Differences in Baseline Dietary Intake and Physical Activity Levels of Adolescents

**DOI:** 10.5888/pcd16.180200

**Published:** 2019-01-03

**Authors:** Renee Euler, Elizabeth Yakes Jimenez, Sarah Sanders, Alena Kuhlemeier, M. Lee Van Horn, Deborah Cohen, Diana Gonzales-Pacheco, Alberta S. Kong

**Affiliations:** 1 Department of Individual, Family and Community Education, University of New Mexico, Albuquerque, New Mexico; 2 Division of Adolescent Medicine, Department of Pediatrics, University of New Mexico Health Sciences Center, Albuquerque, New Mexico; 3 Division of Epidemiology, Biostatistics and Preventive Medicine, Department of Internal Medicine, University of New Mexico Health Sciences Center, Albuquerque, New Mexico; 4 Department of Sociology, University of New Mexico, Albuquerque, New Mexico

## Abstract

**Introduction:**

Differences in dietary intake and physical activity may explain the higher prevalence of obesity among adolescents living in rural versus urban settings. The objective of this cross-sectional secondary analysis was to compare baseline dietary intake and physical activity of adolescents by rurality.

**Methods:**

We analyzed data on 940 adolescents who participated in ACTION PAC (Adolescents Committed to Improvement of Nutrition and Physical Activity), an obesity prevention and management intervention trial conducted from 2014 through 2017 in 8 public high schools in the southwestern United States. Dietary intake was assessed with the Block Food Screener, and participants completed an exercise log and wore an accelerometer to provide data on physical activity. We compared data by rural–urban commuting area (RUCA) codes and log population density by using multilevel models, with students nested within zip code and repeated measures for accelerometer analysis.

**Results:**

After adjusting for socioeconomic status and ethnicity, accelerometer data indicated that moderate-to-vigorous physical activity was 8.17 min/d (*P* = .02) higher and sedentary time was 20.42 min/d (*P* = .02) lower in moderately urban areas than in the urban reference area. Each 1-unit increase in log population density was associated with higher reported intake of whole grains (0.02 ounce equivalents, *P* = .03), potatoes (0.01 cup equivalents, *P* = .02), and added sugar (0.37 tsp, *P* = .02) after adjusting for socioeconomic status and ethnicity.

**Conclusion:**

Differences in reported dietary intake and physical activity level by measures of rurality were small and inconsistent in direction to explain the disparities observed in rural versus urban areas.

## Introduction

One in 5 US adolescents are obese, and nationally representative data indicate that adolescent obesity prevalence is increasing (1–3). Overweight and obese adolescents are at risk for continued obesity and for heart disease, diabetes, cancer, and osteoarthritis as adults, and greater weight gain in early adulthood is associated with greater risk (4,5).

Adolescent obesity is a complex issue. Identifying behavioral, social, and environmental causes is imperative for designing effective obesity prevention and treatment strategies. Rural residency, an environmental factor, is associated with increased prevalence of childhood obesity (5–7). A recent meta-analysis of 10 studies examining urban and rural differences in childhood obesity in the United States found that in a pooled population of more than 74,000 children aged 2 to 19 years, children in rural areas had a 26% greater risk of obesity compared with urban children (8). The 2 studies that reported on adolescents aged 10 to 17 years found a similar difference, with adolescents in nonmetropolitan areas having a 28% greater odds of obesity compared with adolescents in metropolitan areas (6,9). No studies of children or adolescents have examined which environmental factors in rural areas contribute to obesity disparities.

Differences in dietary intake and physical activity levels could potentially explain the higher prevalence of obesity in rural versus urban populations. However, a narrative review of 17 studies examining rural–urban differences in the nutrition and physical activity behaviors of children and adolescents noted inconsistent findings, with few studies examining dietary intake and measuring physical activity using accelerometers (10). In addition, there was substantial variation in the way rurality was defined across studies (10). 

The aim of this study was to compare the baseline dietary intake and physical activity levels of 9th- and 10th-grade public school students in the Southwest who enrolled in the ACTION PAC (Adolescents Committed to Improvement of Nutrition and Physical Activity) intervention trial, by measures of rurality.

## Methods

### Study population

We conducted a cross-sectional secondary analysis of baseline data collected from a subset of participants of ACTION PAC, a cluster-randomized, longitudinal trial of an adolescent obesity prevention and management intervention in school-based health centers (ClinicalTrials.gov identifier NCT02502383). Participants with complete data for the variables of interest were included in the analysis.

Adolescents from 8 public high schools in the Southwest were recruited to participate in the ACTION PAC trial. All participating high schools had functioning school-based health centers and similar food and physical activity environments. All high schools had more than 700 students, of whom more than 40% identified as Hispanic. Participant inclusion criteria were being enrolled in 9th or 10th grade at a participating school and having written informed adolescent assent and parental consent to participate in the longitudinal study. Exclusion criteria were 1) having blood pressure in the range of stage 2 hypertension; 2) having diagnosed diabetes; 3) using corticosteroids, antipsychotics, or medications for the treatment of diabetes, hypertension, or hyperlipidemia; 4) being unable to perform moderate-to-vigorous physical activity (MVPA) or not ambulatory; 5) having a score of 20 or more on the Eating Attitudes Test (11); 6) having developmental disorders that affect weight or ability to understand the study procedures or counseling; and 7) being pregnant. The study protocol was approved by the University of New Mexico Health Sciences Center Human Research Protections Office.

### Data collection

Data were collected at 2 baseline study visits that occurred 1 week apart. Height was measured by using a portable stadiometer (±0.1 cm; Seca Model 213), and weight was measured with a portable electronic scale (±0.1 kg; Seca Model 770). Weight status was determined according to body mass index percentile (12).

Adolescents reported their intake of foods during the past week via the Block Food Screener for Ages 2–17 (2007 version, NutritionQuest). The Block Food Screener estimates average daily intake of fruit and fruit juice (cup equivalents [CEs]); vegetables excluding potatoes and legumes (CEs); whole grains (ounce equivalents [OEs]); legumes (CEs); dairy (CEs); meat, poultry, and fish (OEs); potatoes (CEs); saturated fat (grams); and added sugar (tsp) (13). Reported dietary intake was compared with the 2015–2020 Dietary Guidelines for Americans Recommended Intakes for age (14–18 years) and sex (14).

Physical activity was measured by using the GENEActiv triaxial accelerometer (Activinsights Ltd) for 7 days and the 3-Day Physical Activity Report (3D PAR) (15). Both tools have been validated in children or adolescents, and participants received instructions before use. The accelerometer records movement in acceleration values by using units of gravity (mG, where 1 mG = 0.00981 m/s^2^). An R package (GGIR version 1.5–18) (16–19) was used to reduce accelerometer data to minutes of sedentary and MVPA per day during the hours of 5:00 AM to 11:00 PM. Activity that met the valid wear score generated in GGIR was classified as being sedentary when acceleration was less than 50 mG on average for 60 seconds and as being MVPA when acceleration was above 150 mG. Activity thresholds were chosen on the basis of validation research for the GENEActiv accelerometer (20,21). The 3D PAR was completed for 3 days of the same week, including 1 weekend day and 2 weekdays. Adolescents selected items from 74 predetermined activities or wrote in other activities for every 30-minute block of time between 5:00 AM and midnight (38 total blocks). They also recorded the intensity of the selected activity (light, medium, hard, or very hard) for each block. The blocks were scored by using a standard scoring system (22) that produced total blocks per day spent in MVPA or sedentary activity and total daily metabolic equivalents.

Demographic data, including participant zip code, parental education level, and annual household income, were collected from a health history form completed by the adolescent and parent.

### Measures of rurality

There is no universally recognized classification system or definition of rurality. Commonly used delineations include the Rural–Urban Continuum Codes (RUCCs) (23), Rural–Urban Commuting Area (RUCA) codes (24), and Frontier and Remote Area (FAR) codes (25). This study used both zip code–level RUCA approximation codes developed by the University of Washington Rural Health Research Center (26) and 2010 US Census population density (number of people per square mile) data (25) as measures of rurality.

RUCA codes 1 through 3 are considered metropolitan (urban), codes 4 through 6 are micropolitan, codes 7 through 9 are small town, and code 10 is rural (24). The codes are based on population density, urbanization, and the size and direction of primary daily commuter flow between areas. They are further subdivided on the basis of secondary daily commuter flow size and direction. There is no standard definition or cutoff for population density.

### Data analysis

R version 3.4.3 was used for analysis (19). Relationships with RUCA code and population density were tabulated separately, because each captured substantially unique variance. RUCA codes explain about 23% of the variation in log population density. Four RUCA codes were present in the data: 1.0 (metropolitan area core: primary commuting flow within an urbanized area), 2.0 (metropolitan area high commuting: primary flow of ≥30% to an urbanized area), 2.1 (metropolitan area high commuting: secondary flow of 30%–50% to a larger urbanized area), and 10.0 (rural areas: primary commuting flow to a tract outside an urbanized area or urban cluster). Because so few participants were represented in RUCA code 10.0 (n = 3), they were dropped from analysis. The cutoff for rural versus urban population density was set at fewer than 1,000 people per square mile for descriptive analyses (27); the continuous variable log population density was used in all models.

Because measures of rurality were determined at the zip code level, there were multiple respondents per zip code, and multilevel models in which participants were nested within zip code were used for all analyses. For the accelerometer data, 3-level repeated measures models were used (ie, day nested within participant nested within zip code). To account for nonwear of accelerometers, each day was weighted by the proportion of nonwear, and all available data were included in the analysis. To examine the overall relationship between RUCA code and reported dietary intake and physical activity, we conducted 2-degrees-of-freedom log-likelihood (LL) ratio tests, which examine whether the RUCA codes were associated together with each outcome. Two dummy variables represented RUCA codes at the 3 included levels (1.0, 2.0, 2.1), with 1.0 as the reference category. If we found an overall effect of RUCA code, differences in means between the reference category and RUCA codes 2.0 and 2.1 were interpreted. We controlled for socioeconomic status as measured by annual family income (reference group, ≥$20,000 per year) and parental education level (reference group, <high school graduation). A difference in LL ratio test was used to test whether RUCA code contributed to variation in reported dietary intake and physical activity beyond the socioeconomic variables. LL tests for fixed effects were conducted by using maximum likelihood estimation.

Each of the dietary and physical activity outcomes was separately regressed on log population density, alone and then including the socioeconomic variables. In these analyses the hypotheses were assessed directly by the regression parameter for log density and no LL ratio test was needed. Intra-class correlation coefficients were tabulated to describe the extent to which participants’ reported dietary intake and physical activity differed within their zip codes or across zip codes. We did not tabulate results on participant weight status, because the design and recruitment methods of the longitudinal trial influenced the prevalence of overweight and obesity; approximately 40% of participants were overweight or obese at baseline by design.

## Results

### Participant characteristics

Most participants (80%) lived in RUCA code 1.0, identified as Hispanic (86%), and were female (55%) (Table 1). The proportion of Hispanic participants increased with less urban RUCA codes, and the proportion of female participants decreased with less urban RUCA codes. Participants from RUCA code 2.0 (moderately urban) had the highest proportion of annual family income less than $20,000 (54%) and the lowest proportion of parents who were college graduates (8%). Differences using the population density cutoff of 1,000 people per square mile were inconsistent with RUCA code observations; less densely populated areas (<1,000 people/mile^2^) had a higher proportion of female participants, a lower proportion of families with income less than $20,000 per year, and a higher proportion of parents who were college graduates. 

**Table 1:** Characteristics of Participants, by Measures of Rurality^a^, Study of Rural–Urban Differences in Baseline Dietary Intake and Physical Activity Levels Among Adolescents, ACTION PAC Cluster-Randomized Trial

Characteristic	Total (N = 940)	Zip Code–Level RUCA Code^b^			Population Density	
		1.0 (n = 749)	2.0 (n = 144)	2.1 (n = 47)	≥1,000 People/mile^2^ (n = 396)	<1,000 People/mile^2^ (n = 510)
**Age, mean (SD), y**	15.3 (0.7)	15.4 (0.7)	15.1 (0.7)	15.2 (0.7)	15.5 (0.7)	15.2 (0.7)
**Female sex**	519 (55)	419 (56)	75 (52)	25 (53)	210 (53)	291 (57)
**Hispanic ethnicity**	810 (86)	628 (84)	136 (94)	46 (98)	339 (86)	439 (86)
**Race^c^,**						
White	119 (13)	111 (15)	7 (5)	1 (2)	45 (11)	70 (14)
Black	34 (4)	34 (5)	0	0	16 (4)	18 (4)
American Indian	26 (3)	25 (3)	1 (1)	0	18 (5)	7 (1)
Asian	6 (0)	5 (1)	1 (1)	0	5 (1)	1 (0)
Pacific Islander	1 (0)	1 (0)	0	0	1 (0)	0
Multiple	24 (2)	22 (3)	1 (1)	1 (2)	15 (4)	9 (2)
**Annual household income^d^, $**						
<20,000	392 (42)	300 (40)	78 (54)	14 (30)	182 (46)	196 (38)
≥20,000	548 (58)	449 (60)	66 (46)	33 (70)	214 (54)	314 (62)
**Parent/guardian education level^e^**						
Less than high school graduate	291 (31)	219 (29)	53 (37)	19 (40)	135 (34)	150 (29)
High school graduate or some college	506 (54)	403 (54)	79 (55)	24 (51)	214 (54)	271 (53)
College graduate	143 (15)	127 (17)	12 (8)	4 (9)	47 (12)	89 (18)

Abbreviations: ACTION PAC, Adolescents Committed to Improvement of Nutrition and Physical Activity; RUCA, rural–urban commuting area; SD, standard deviation.

a Values are no. (%) unless otherwise indicated.

b 1.0 = Metropolitan area core: primary commuting flow within an urbanized area; 2.0 = Metropolitan area high commuting: primary flow 30% or more to an urbanized area; 2.1 = Metropolitan area high commuting: secondary flow 30% to 50% to a larger urbanized area.

c Seventy-eight percent of participants selected only an ethnicity.

d Four percent of participants had missing data for family income.

e One percent of participants had missing data for parent/guardian education level.

Overall reported diet quality was poor; 73% to 99% of adolescents reported that they consumed less than the 2015–2020 Dietary Guidelines for Americans sex- and age-specific recommended intake of fruits, vegetables, whole grains, dairy, and legumes. Less than 1% of participants met the recommended intake of legumes, and more participants met the recommendations for fruit than for all other food groups.

### Differences in dietary intake and physical activity level by RUCA code

For most dietary intake variables and for blocks of MVPA and sedentary time on the 3D PAR, we found no significant relationship with RUCA code overall (Table 2). We found a significant overall relationship with RUCA code for whole grains (*P* = .02) and minutes per day of MVPA (*P* = .02) and of sedentary time (*P* = .02) as measured by accelerometer. The overall relationship with whole grains did not persist after controlling for family income, parent education level, and ethnicity. As measured by accelerometer, MVPA and sedentary time were 8.71 min/d (*P* = .02) higher and 20.42 min/d (*P* = .02) lower in RUCA code 2.0 than in RUCA code 1.0 after controlling for socioeconomic status and ethnicity. Minutes per day of MVPA and sedentary time for RUCA 2.1 fell in between and were not significantly different from the minutes per day for the other RUCA codes.

**Table 2:** Differences in Reported Dietary Intake and Physical Activity^a^ by Zip Code–Level Rural–Urban Commuting Area (RUCA) Codes, Study of Rural–Urban Differences in Baseline Dietary Intake and Physical Activity Levels Among Adolescents, ACTION PAC Cluster-Randomized Trial

Variable	Unadjusted Results				Adjusted Results							
	Intercept (SE)	Zip Code–Level RUCA Code^b^		LL Test^c^ (*P* Value)	Intercept (SE)	Zip Code–Level RUCA Code^b^		Annual Household Income <$20,000 (SE)	Parent Education		Hispanic (SE)	LL Test^c^ (*P* Value)
		2.0 (SE)	2.1 (SE)			2.0 (SE)	2.1 (SE)		<High School (SE)	High School Graduate or Some College (SE)		
**Dietary intake as estimated by Block Food Screener (N = 940)**												
Fruit/fruit juice, CE	1.44 (0.04)	−0.21 (0.10)	−0.02 (0.16)	4.49 (.11)	1.49 (0.12)	−0.21 (0.10)	−0.02 (0.16)	0.07 (0.09)	−0.02 (0.13)	−0.04 (0.11)	−0.09 (0.05)	4.34 (.11)
Vegetables^d^, CE	0.72 (0.02)	−0.07 (0.05)	0.08 (0.08)	3.40 (.18)	0.85 (0.06)	−0.05 (0.05)	0.10 (0.08)	−0.03 (0.05)	−0.06 (0.06)	−0.03 (0.06)	−0.09 (0.05)	3.17 (.21)
Legumes, CE	0.14 (0.01)	0.02 (0.02)	0.05 (0.03)	2.69 (.26)	0.11 (0.02)	0.02 (0.02)	0.04 (0.03)	0.01 (0.02)	0.02 (0.02)	0.02 (0.02)	0.04 (0.02)	1.98 (.37)
Whole grains, OE	0.52 (0.02)	−0.11 (0.04)	−0.09 (0.07)	7.47 (.02)	0.61 (0.05)	−0.10 (0.04)	−0.07 (0.07)	−0.05 (0.04)	−0.05 (0.05)	−0.04 (0.05)	−0.02 (0.05)	5.33 (.07)
Meat/poultry/fish, OE	2.73 (0.12)	−0.07 (0.32)	0.55 (0.43)	1.67 (.44)	3.01 (0.29)	−0.02 (0.34)	0.61 (0.45)	−0.02 (0.22)	−0.21 (0.31)	0.04 (0.28)	−0.28 (0.26)	1.87 (.39)
Dairy, CE	1.32 (0.04)	0.06 (0.09)	0.13 (0.15)	1.23 (.54)	1.42 (0.10)	0.08 (0.09)	0.15 (0.15)	−0.08 (0.08)	−0.03 (0.12)	−0.02 (0.10)	−0.03 (0.10)	1.62 (.45)
Potato, CE	0.31 (0.01)	−0.06 (0.03)	−0.04 (0.04)	4.37 (.11)	0.33 (0.03)	−0.05 (0.03)	−0.03 (0.04)	−0.02 (0.02)	0.08 (0.03)	0.07 (0.03)	−0.08 (0.03)	3.55 (.17)
Saturated fat, g	18.31 (0.49)	−0.65 (1.24)	2.24 (1.92)	1.76 (.41)	19.42 (1.34)	−0.47 (1.32)	2.44 (1.96)	−0.75 (1.04)	0.14 (1.48)	0.32 (1.31)	−1.01 (1.24)	1.79 (.41)
Added sugar, tsp	8.36 (0.26)	−1.04 (0.64)	1.03 (1.06)	3.71 (.16)	7.45 (0.75)	−1.17 (0.65)	0.88 (1.06)	0.04 (0.58)	1.45 (0.83)	0.79 (0.74)	0.03 (0.70)	4.34 (.11)
**3-Day physical activity record (N = 795)**												
Total activity, MET	75.24 (0.68)	2.31 (1.66)	6.00 (2.85)	5.30 (.07)	75.72 (1.49)	2.37 (1.87)	5.84 (2.94)	6.25 (2.87)	−0.62 (2.20)	0.26 (1.96)	6.25 (2.87)	5.50 (.06)
MVPA, 30-min block	5.56 (0.13)	0.26 (0.33)	0.41 (0.56)	1.03 (.60)	5.59 (0.38)	0.26 (0.33)	0.34 (0.60)	0.44 (0.57)	0.11 (0.43)	0.18 (0.39)	0.44 (0.57)	1.10 (.58)
Sedentary, 30-min block	29.41 (0.17)	−0.08 (0.43)	−0.25 (0.64)	0.62 (.92)	29.68 (0.42)	−.004 (0.49)	−0.04 (0.64	−0.24 (0.63)	−0.16 (0.48)	−0.23 (0.42)	−0.24 (0.63)	.15 (.93)
**Accelerometer data (N = 891)**												
MVPA, min/d	52.61 (1.14)	8.10 (2.85)	3.42 (4.92)	7.59 (.02)	53.71 (3.29)	8.17 (2.87)	3.78 (4.93)	4.65 (2.61)	−5.31 (3.71)	−1.94 (3.29)	−2.10 (.09)	7.71 (.02)
Sedentary, min/d	818.25 (2.79)	−22.13 (7.00)	−6.28 (11.95)	7.70 (.02)	830.46 (7.93)	−20.42 (6.91)	−5.29 (11.90)	−14.17 (6.30)	5.33 (8.95)	−2.52 (7.95)	−3.02 (7.46)	7.62 (.02)

Abbreviations: ACTION PAC, Adolescents Committed to Improvement of Nutrition and Physical Activity; CE, cup equivalent; LL, log-likelihood ratio; MET, metabolic equivalent of task, MVPA, moderate to vigorous physical activity; OE, ounce equivalent; RUCA, rural–urban commuting area; SE, standard error.

a Dietary intake was measured by using the Block Food Screener for ages 2–17 (2007 version, NutritionQuest) (13). Physical activity was measured by using the GENEActiv triaxial accelerometer (Activinsights Ltd) for 7 days and the 3-Day Physical Activity Report (3D PAR) (15).

b 1.0 = Metropolitan area core: primary commuting flow within an urbanized area (reference group); 2.0 = metropolitan area high commuting: primary flow 30% or more to an urbanized area; 2.1 = metropolitan area high commuting: secondary flow 30% to 50% to a larger urbanized area.

c Multilevel models in which participants were nested within zip code were used for all analyses. For the accelerometer data, 3-level repeated measures models with day nested within participant nested within zip code were used. To examine the overall relationship between RUCA code and reported dietary intake and physical activity, 2-degrees-of-freedom LL tests, which examine whether together the 3 RUCA codes predicted each outcome, were used.

d Vegetables not including potatoes or legumes.

### Differences in dietary intake and physical activity level by log population density

Each 1-unit increase in log population density was associated with increases in reported intake of whole grains (0.02 OE, *P* = .03), potatoes (0.01 CE, *P* = .02), and added sugar (0.37 tsp, *P* = .02), after adjustment for socioeconomic status and ethnicity (Table 3). We found no significant relationship between other dietary intake variables or any of the physical activity variables and log population density. Overall, intraclass correlation coefficients indicated that more than 98% of the variation in the sample with respect to reported dietary intake and physical activity occurred within zip codes as opposed to between zip codes.

**Table 3:** Differences in Reported Dietary Intake and Physical Activity,^a^ by Log Population Density, Study of Rural-Urban Differences in Baseline Dietary Intake and Physical Activity Levels Among Adolescents, ACTION PAC Cluster-Randomized Trial

Variable	Unadjusted Results				Adjusted Results						
	Intercept (SE)	Log Population Density (SE)	*P* b	ICC	Intercept (SE)	Log Population Density (SE)	Family Income <$20K (SE)	Parent Education		Hispanic (SE)	*P* b
								<High School (SE)	High School Graduate or Some College (SE)		
**Dietary intake as estimated by Block Food Screener (N = 906)**											
Fruit/fruit juice, CE	1.19 (0.14)	0.03 (0.02)	.13	0	1.28 (0.18)	0.03 (0.02)	0.02 (0.09)	−0.01 (0.13)	−0.03 (0.12)	−0.10 (0.11)	.15
Vegetables,^c^ CE	0.76 (0.08)	−0.01 (0.01)	.63	0.005	0.87 (0.09)	−0.003 (0.01)	−0.03 (0.05)	−0.07 (0.07)	−0.03 (0.06)	−0.08 (0.06)	.77
Legumes, CE	0.17 (0.03)	−0.01 (0.01)	.33	0.019	0.12 (0.04)	−0.01 (0.01)	0.01 (0.02)	0.02 (0.02)	0.02 (0.02)	0.04 (0.02)	.23
Whole grains, OE	0.38 (0.06)	0.02 (0.01)	.07	0.002	0.47 (0.08)	0.02 (0.01)	−0.06 (0.04)	−0.06 (0.06)	−0.05 (0.05)	−0.03 (0.05)	.03
Meat/ poultry/ fish, OE	2.53 (0.44)	0.04 (0.07)	.56	0.014	2.80 (0.52)	0.04 (0.07)	0.01 (0.23)	.029 (0.23)	−0.01 (0.28)	−0.24 (0.27)	.57
Dairy, CE	1.40 (0.13)	−0.01 (0.02)	.62	0	1.46 (0.16)	−0.01 (0.02)	−0.05 (0.08)	−0.05 (0.12)	−0.04 (0.11)	−0.01 (0.10)	.73
Potato, CE	0.21 (0.04)	0.02 (0.01)	.02	0.002	0.25 (0.05)	0.01 (0.01)	−0.03 (0.02)	.07 (0.03)	0.07 (0.03)	−0.08 (0.03)	.02
Saturated fat, g	16.95 (1.78)	0.23 (0.28)	.42	0.003	17.98 (2.21)	0.24 (0.29)	−0.57 (1.07)	−0.25 (1.05)	0.04 (1.35)	−0.82 (1.26)	.41
Added sugar, tsp	5.77 (0.93)	0.40 (0.15)	.01	0	4.99 (1.16)	0.37 (0.15)	−0.07 (0.60)	1.50 (0.85)	0.85 (0.76)	0.08 (0.71)	.02
**3-Day physical activity record (N = 764)**											
Total activity, MET	80.81 (2.80)	−0.79 (0.44)	.08	0.007	81.21 (3.34)	−0.83 (0.44)	1.65 (1.60)	−1.34 (2.27)	−0.53 (2.03)	−0.47 (1.92)	.07
MVPA, 30-min block	6.06 (0.56)	−0.07 (0.09)	.41	0.007	6.20 (0.66)	−0.09 (0.09)	0.28 (0.32)	−0.09 (0.45)	0.02 (0.40)	−0.27 (0.38)	.34
Sedentary, 30-min block	29.06 (0.64)	0.05 (0.10)	.60	0.010	29.15 (0.72)	0.07 (0.10)	−0.46 (0.35)	.04 (0.49)	−0.08 (0.44)	0.14 (0.41)	.45
**Accelerometer data (N = 860)**											
MVPA, min/d	59.78 (4.48)	−0.93 (0.70)	.19	—d	59.72 (5.28)	−1.07 (0.69)	4.99 (2.71)	−3.62 (3.83)	−0.33 (3.41)	−1.58 (3.16)	.13
Sedentary, min/d	805.87 (12.09)	1.41 (1.89)	.46	—d	817.68 (13.33)	1.91 (1.77)	−13.85 (6.54)	1.56 (9.23)	−6.42 (8.21)	−2.63 (7.61)	.29

Abbreviations: ACTION PAC, Adolescents Committed to Improvement of Nutrition and Physical Activity; CE, cup equivalents; ICC, intraclass correlation coefficient; MET, metabolic equivalent of task; MVPA, moderate to vigorous physical activity; OE, ounce equivalents; SE, standard error.

a Dietary intake was measured by using the Block Food Screener for ages 2–17 (2007 version, NutritionQuest) (13). Physical activity was measured by using the GENEActiv triaxial accelerometer (Activinsights Ltd) for 7 days and the 3-Day Physical Activity Report (3D PAR) (15).

b Multilevel models in which participants were nested within zip code were used for all analyses. For the accelerometer data, 3-level repeated measures models with day nested within participant nested within zip code were used. In these analyses, the hypotheses were assessed directly by the regression parameter for log density.

c Vegetables not including potatoes or legumes.

d Because there are multiple ICCs for analyses with 3 levels, we reported the variance components instead. The variance for zip code is 3.44 for MVPA, 749.97 for individual, and 1332.47 for time within individual; for sedentary behavior, the variances are 63.75, 4110.56, and 9298.99, respectively.

## Discussion

Overall, differences in reported dietary intake and physical activity level by RUCA code and log population density were small and not entirely consistent with the hypothesis that differences in dietary intake and physical activity have a prominent role in explaining observed rural versus urban obesity disparities in adolescents. The observed differences mostly persisted after controlling for socioeconomic status and ethnicity, indicating that perhaps other community level access factors were driving the differences. For example, the observed relationships between dietary intake and population density could reflect increased access to grocery stores (whole grains) and fast food restaurants (potatoes, added sugar) in more densely populated areas.

Our finding of a few significant differences in dietary intake by measures of rurality is consistent with a recent narrative review (10). We found little consistency in observed differences in food groups or nutrients by measures of rurality across 5 studies assessing dietary intake in US children or adolescents by measures of rurality (10). In the 3 studies that included adolescents, one found no significant differences in dietary intake between urban and rural adolescents (5). The second study noted a slightly smaller percentage of rural adolescents (12.2%) than urban adolescents (16.5%) who reported consuming 2 or more cups of fruit per day (27), and the third study found that nonmetropolitan and metropolitan black youth consumed fatty snack foods more often than did white metropolitan youth (28).

Our observation that physical activity level was higher with decreasing urbanization is also consistent with a recent narrative review, which found that urban youth were less active than rural youth in 9 of 16 studies examining physical activity levels in US children or adolescents by measures of rurality (10). The only 2 studies to use accelerometers, both conducted by Moore et al, had inconsistent findings, noting that MVPA was higher among urban middle school students than among rural middle school students in the southeastern United States (29) and that MVPA was higher in rural 4th- through 8th-grade girls compared with suburban and urban girls, but not boys, in North Carolina (30). Observed inconsistencies may reflect regional differences in physical activity infrastructure and employment and recreational opportunities for physical activity by measures of rurality or differences in how rurality is defined. In an examination of determinants of physical activity, both rural and urban families expressed the following as barriers: physical distance to activity areas, cost, electronic media, safety concerns, and the need for parental supervision (31). Some of the observed differences in our study could be related to access to electronic media and the internet. Internet connectivity is less reliable and less available in more rural settings and, conversely, ubiquitous in urban settings. This fact is supported by our finding that sedentary time was highest in youth from the most urban areas. We found inconsistent results for physical activity using RUCA codes versus log population density in our analysis.

Our study has several strengths, including a large sample size, the reporting of both dietary intake and physical activity data, and the objective measure of physical activity through the use of accelerometers. Our study also has limitations. First, the development patterns of the western US make it difficult to differentiate between subtle variations in rurality that may affect access to health care and nutrition services and the food and physical activity environment. Research staff members who observed the actual settings were surprised to find little variation in RUCA codes among participants in the study. Almost all of the communities involved were considered metropolitan/urban based on RUCA code, but the most urban community had many grocery stores and fast-food restaurants with easy access to the schools, compared with another community that had only 1 restaurant. Although zip code–level RUCA codes and population density were both used in this study, there are additional measures of rurality, and ours may not reflect differences within the zip code–level RUCA codes. For example, some of the communities involved in the study are health professional shortage areas, while others are not. Findings for RUCA code 2.1 should be interpreted cautiously, because this group included fewer than 50 participants. Dietary intake was assessed by using a food frequency screener, which allowed us to report on a limited set of variables. Use of a full food frequency questionnaire could have provided a more comprehensive, and potentially accurate, picture of dietary intake. Both the food frequency screener and the physical activity record have limitations related to social desirability bias, although we would not expect the magnitude of the bias to vary by measures of rurality. Finally, participants in this study were public school students who chose to participate in a longitudinal obesity prevention and management intervention trial, limiting the generalizability of the results.

Our findings have public health implications. The reported baseline dietary intake of all adolescents in the study was inadequate compared with the Dietary Guidelines for Americans, indicating an ongoing need for nutrition education and for policies that support access to nutritious foods in schools and communities. Given the inconsistent findings related to differences in dietary intake and physical activity in children and adolescents by measures of rurality, additional research is needed to understand the underlying causes of rural–urban obesity disparities. Future research should be conducted with representative rural and urban populations and include standardized, appropriate measures of rurality; comprehensive measures of dietary intake; objective measures of physical activity, such as accelerometers; and assessment of additional environmental and health system factors that could be causing obesity disparities among adolescents.
